# Nudifloside, a Secoiridoid Glucoside Derived from Callicarpa nudiflora, Inhibits Endothelial-to-Mesenchymal Transition and Angiogenesis in Endothelial Cells by Suppressing Ezrin Phosphorylation

**DOI:** 10.7150/jca.91566

**Published:** 2024-03-11

**Authors:** Dongliang Zhuo, Yinlong Mei, Chaozhan Lin, Aizhi Wu, Yuehua Luo, Hong Lu, Jianjiang Fu

**Affiliations:** 1 School of Pharmacy, Jiangxi University of Chinese Medicine, Nanchang 330004, China.; 2 Institute of Clinical Pharmacology, Guangzhou University of Chinese Medicine, Guangzhou 510006, China.; 3 Jiangxi Provincial Institute for Drug Control, Nanchang, 330029, China.; 4 Network and Educational Technology Center, Jiangxi University of Chinese Medicine, Nanchang 330004, China.; 5 Jiangxi Province Key Laboratory for Diagnosis, Treatment, and Rehabilitation of Cancer in Chinese Medicine, China.

**Keywords:** Nudifloside, EndoMT, angiogenesis, Ezrin, *Callicarpa* nudiflora.

## Abstract

*Callicarpa nudiflora* is a traditional folk medicine in China used for eliminating stasis to subdue swelling. Several compounds from *Callicarpa nudiflora* have been proved to show anti-inflammatory, haemostasis, hepatitis, and anti-proliferative effects. Tumor endothelial cells play crucial roles in tumor-induced angiogenesis. Recently, it was demonstrated that ECs may be the important source of cancer associated fibroblasts (CAFs) through endothelial to mesenchymal transition (EndoMT). In this study, we evaluated the effects of nudifloside (NDF), a secoiridoid glucoside from Callicarpa Nudiflora, on TGF-β1-induced EndoMT and VEGF-induced angiogenesis, and the underlying mechanisms were also involved. It was found that NDF significantly inhibited enhanced migration, invasion and F-actin assembly in endothelial cells (ECs) exposed in TGF-β1. NDF obviously reversed expression of several biomarkers associated with EndoMT and recovered the morphological characteristics of ECs and tube-like structure induced by TGF-β1. Furthermore, treatment of NDF resulted in a significant destruction of VEGF-induced angiogenesis *in vitro* and ex vivo. Data from co-immunoprecipitation assay provided the evidence that Ezrin phosphorylation and the interaction with binding protein can be inhibited by NDF, which can be confirmed by data from Ezrin silencing assay. Collectively, the application of NDF inhibited TGF-β1-induced EndoMT and VEGF-induced angiogenesis in ECs by reducing Ezrin phosphorylation.

## Introduction

Endothelial to mesenchymal transition (EndoMT) descripts a dynamic trans-differentiation program of endothelial cells (ECs), in which ECs lose their endothelial traits, such as cell-cell junction and angiogenic ability, and acquire characteristics of mesenchymal cells, such as invasion, migration and proliferation 1. Furthermore, during EndoMT, several characteristic endothelial markers, such as VE-cadherin, CD31/PECAM-1, TIE1, TIE2, and von Willebrand Factor (vWF), disappear, while certain mesenchymal biomarkers, including SMA, SM22a, CD44, N-Cadherin, vimentin, I/III-COL, and FSP-1 etc, emerge 2, 3. Initially, this process was found during embryogenesis 2. In past decades, the pathological contributions of EndoMT were reported in many diseases, including cardiac fibrosis, pulmonary arterial hypertension, atherosclerosis 3. Recently, emerging evidence has demonstrated that EndoMT plays important roles in cancer development 4, 5. According to the finding of Wawro and colleagues 6, originating from endothelium through EndoMT may be an important way of cancer associate fibroblasts (CAFs) production, and this phenomenon was also demonstrated by other groups 7, 8. In contrast, Wei et al. more recently demonstrated that CAF-derived PAI-1 can also promote EndoMT of lymphatic ECs and contribute to the lymphatic metastasis of cervical squamous cell carcinoma 9.

As the primary constructers of blood vessels, ECs play a pivotal role during the development of cancer 10, 11. Upon stimulated by cytokines, chemokines and growth factors released from cancer cells, ECs become activated and facilitate proliferation, migration, degradation of extracellular matrix (ECM), as well as formation of new vessels from pre-existing one. These collective processes ultimately result in tumor development 12. In addition, tumor angiogenesis also plays an important role in the formation of metastatic foci. Unlike regular vessels, tumor blood vessels exhibit reduced pericytes and basement membrane, as well as smaller diameter, which may facilitate migration and invasion of cancer cells 13.

**Figure 1:**
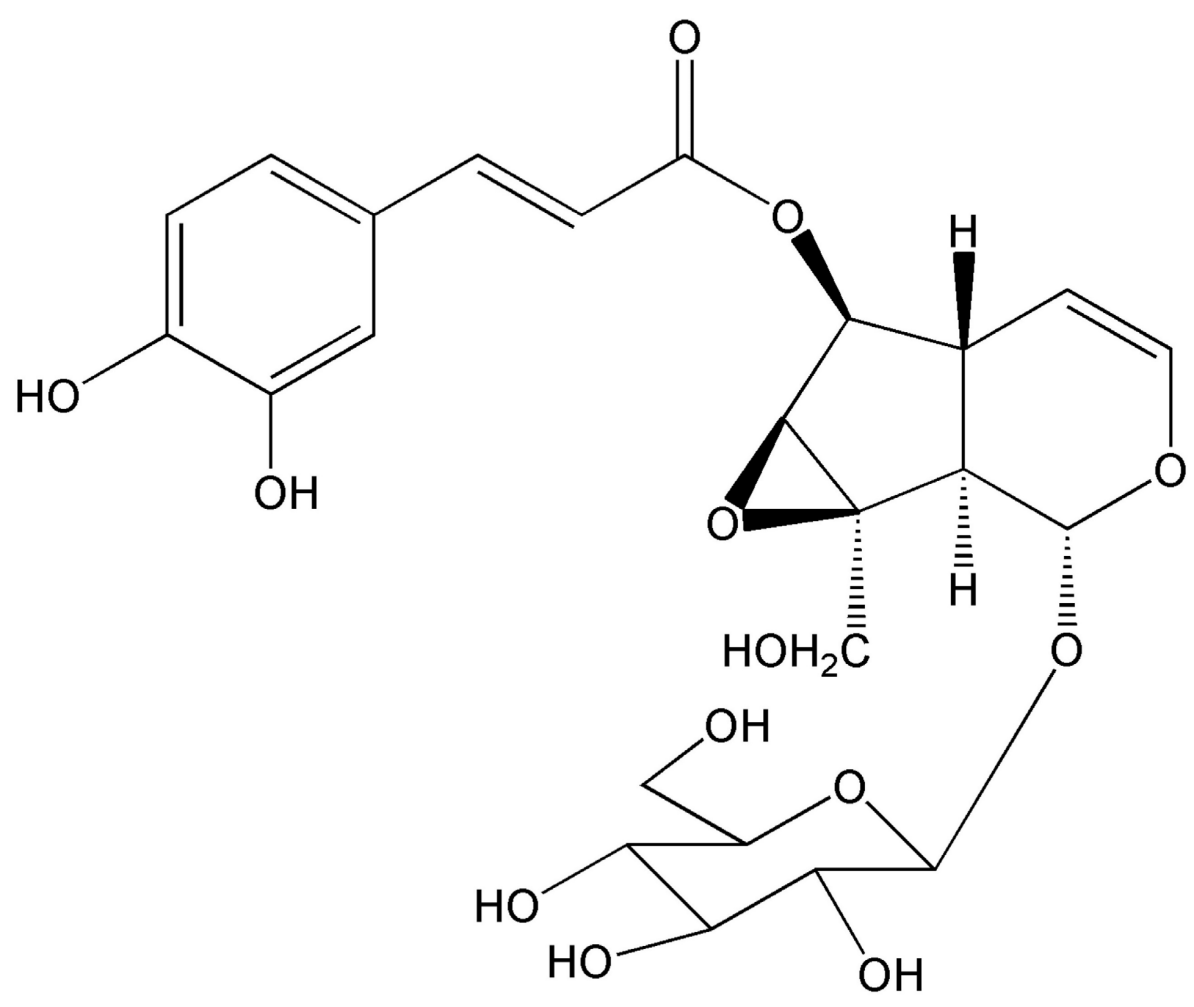
The molecular structure of nudifloside.

The interests of our group were focused on screening of leading compounds derived from natural products against cancer metastasis. By anti-migratory-guiding fractionation, it was discovered that nudifloside (NDF), which was isolated from leaves of *Callicarpa nudiflora* (molecular structure of NDF is shown in Fig. 1) 14, exhibited significant inhibition on TGF-β1-induced migration in ECs. Given that TGF-β1 is the most important inducer of EndoMT, we then hypothesized that NDF may also possess the inhibitory effects on EndoMT. Therefore, the effects of NDF on EndoMT were evaluated, and the underlying mechanisms of its action were also explored. In addition, we also discussed the anti-angiogenetic properties of NDF.

## Materials and methods

### Materials

NDF (purity ≥ 95%) is product of APExBIO Technology LLC (Houston, Texas). VEGF_165_ (HY-P7110A) and TGF-β1(HY-P78168) were purchased from MedChemExpress LLC (Shanghai, China). PTK787 (S1101) and TGF-βRI inhibitor SD208 (S7624) were purchased from Selleck (Shanghai, China). Alexa Fluor™ 488 Phalloidin (A12379) was purchased from ThermoFisher Scientific (Shanghai, China). Several primary antibodies were used in present study. Antibodies of Ezrin (ab40839), p-Ezrin (phosphor T567, ab47293), PODXL (ab150358), CD31 (ab9498), α-SMA (ab7817), I-Col(ab138492), III-Col (ab184993), β-Actin (ab8226), VE-Cadherin (ab3136320 and ICAM-1 (ab282575) were the products of Abcam.

### Cell lines and animals

Human Umbilical Vein Endothelial Cells (HUVECs) were purchased from FuDan IBS Cell Center (Shanghai, China) and maintained in M199 medium (Gibco, Carlsbad, CA) supplemented with 20% FBS and 1% pen/strep in 5% CO_2_ at 37 °C. Another human endothelial cells EA.hy 926 cells, obtained from American Type Culture Collection (Manassas, VA, USA), were cultured in Dulbecco's Modified Eagle's Medium (DMEM) supplemented with 10% FBS, 2 mM L-glutamine, 1% pen/strep in 5% CO_2_ at 37 °C.

For rat aortic ring assay, specific pathogen-free Sprague-Dawley rats aged 5-7 weeks old were obtained from Laboratory Animal Science and Technology Center, Jiangxi University of Traditional Chinese Medicine. Rats were allowed for ad libitum access for water and food.

### Ethics statement

All the procedures for animal using were performed in strict accordance with the protocol approved by Animal Ethics Committee, Jiangxi University of Traditional Chinese Medicine.

### Tube formation assay

96-well plates were coated with 40 μl Matrigel and incubated for 30 min at 37 °C to promote solidification, serum deprived ECs (4×10^4^) were seeded on the top of Matrigel and incubated in 200 μl medium contained NDF at different concentrations in the presence or absence of TGF-β1 or/and VEGF. After 48 h incubation, the capillary tubes were captured by an inverted microscope (Leica DMI 3000B, Germany), and quantification of total lengths of the completed tubule structure was measured by ImageJ in pixel.

### Cell migration assay

Cell migration assay was performed in AP 48 chamber system (Neuro Probe). Briefly, 10 μg of fibronectin was evenly spread on the lower surface (rough surface) of polycarbonate membrane (8-μm pore size). 4×10^5^ of ECs were added into each well of the upper chambers. 30 μl growth medium with 10% FBS and 10% Collagen I were added into each well of the lower compartments. After 24 h incubation, the migrated cells on the lower surface of polycarbonate membrane were washed by PBS, fixed and stained by 0.5% crystal violet. Images were captured under an inverted microscope (Leica DMI 3000B, Germany).

### Cell invasion assay

ORIS^TM^ cell invasion assay system (Platypus Technologies, Madison, WI) was used. Briefly, Serum deprived (24 h) ECs (5×10^5^ per well) were seeded into collagen-coated plate with a stopper. After 24 h incubation, the stopper was removed and a cell-exclusive zone was revealed in each well. Then the cells were washed and stained by Calcein AM (final concentration 0.5 μg/ml) for 40 min. The images were captured under an inverted fluorescence microscope (Leica DMI 3000B, Germany). After 3 times washing, the cells were treated with NDF for 24 h. The ECs were stained by Calcein AM again to evaluate the invasion of cells into an exclusion zone.

### Labeling of F-actin

ECs were seeded into 8-well Chamber Slide (ibidi GmbH, Germany) at a density of 1×10^3^ per well. After 24h-incubation, the cells were treated by indicated NDF for another 24h. After that, the cells were washed, fixed and permeabilized with 0.5% Triton X-100 for 5 min. To visualize F-actin, Alexa Fluor™ 488 Phalloidin diluted in cytoskeleton buffer with sucrose (CBS) was used. After one-hour incubation in dark, the cellular nuclei were stained using 4',6-diamidino-2-phenylindole (DAPI, Sigma-Aldrich, USA), and imaged under a fluorescence microscopy (Olympus BX-63, Japan).

### Cell Immunofluorescent analysis

1×10^3^ cells were plated into 8-well Chamber Slide. After 24-hour incubation, indicated concentration of NDF were added for 24 hours. Then the cells were fixed, permeabilized and blocked. VE-cadherin (1:100) or α-SMA (1:50) antibodies were put into plate for 1 hour incubation, and then the cells were incubated with Alexa Fluor® 488 conjugated IgG (1:100 dilution) for 1 h at room temperature. After washing in PBS, the cells were mounted by ProLong Gold anti-fade reagent with DAPI (CellSignaling Technology). The fluorescent pictures were captured under a fluorescence microscopy (Olympus BX-63, Japan).

### Rat aortic ring angiogenesis assay

The thoracic aorta, isolated from a freshly sacrificed Sprague-Dawley rat, was cut into 1-1.5 mm ring sections under cold and sterile condition. Then, these ring sections were carefully removed into 24-well plates pre-coated by 150 μL Matrigel. Another 150 μL Matrigel was added into each well. After solidification, the aortic rings were incubated in 37℃ CO_2_ incubator for 14 days at presence of 10 ng/ml VEGF. Microvessels were fixed and captured by an inverted microscope (Leica DMI 3000B, Germany). Three independent experiments were performed for each treatment.

### Co-immunoprecipitation

Co-immunoprecipitation was carried out previously described 15. Log-phase endothelial cells were treated with or without NDF for 24 h. The treated cells were collected and lysed with ice cold lysis buffer at 4 °C for 30 min. After centrifugation, the supernatants were collected and mixed with antibody-conjugated beads. The mixtures were incubated and rotated for 12 h at 4℃ on a tube rotator. After washed with ice-cold lysis buffer for three times, the co-IP samples were mixed with 6 × sample buffer. The co-precipitated proteins were separated by 10% SDS-PAGE and checked by immunoblotting according to Burckhardt's protocol.

### Ezrin silencing procedure mediated by Small interfering RNA (siRNA)

RNA interference was involved in present study to silence Ezrin gene in ECs. Log-phase ECs were harvested and seeded in a 75 cm^2^ culture flask at density of 2×10^7^. When 70-80% confluency was reached, Ezrin siRNA (SignalSilence® Ezrin siRNA I, CellSignaling Technology) was added at a final concentration of 100 nM by siRNA Transfection Reagent (sc-29528; Santa Cruz Biotechnology). After 24 h transfection, cells were harvested for further experiments.

### Western blotting analysis

Total protein was isolated from ECs treated by indicated NDF using RIPA buffer (Beyotime, Shanghai, China). The protein concentration was measured by a BCA Protein Assay Kit (Beyotime, Shanghai, China). The extracted proteins were separated by SDS-PAGE and then transferred to PVDF membranes. After transfer, the membranes were blocked by 5% BSA and incubated with primary antibodies and corresponding secondary antibodies by turn. Following three times washing, the membranes were developed using an enhanced chemiluminescence kit (Millipore) to visualize the target proteins. ImageJ software was involved to semi-quantified resulting bands.

### Statistical analysis

All values are expressed as mean ± S.D. All statistical analysis were carried out by GraphPad Prism (Version 4.5; La Jolla, CA). Comparisons between different groups and statistical significance were calculated by one-way analysis of variance (the one-way ANOVA). P˂0.05 was regarded as a statistically significant difference.

## Results

### Increased cell motility stimulated by TGF-β1 can be suppressed by NDF

**Figure 2:**
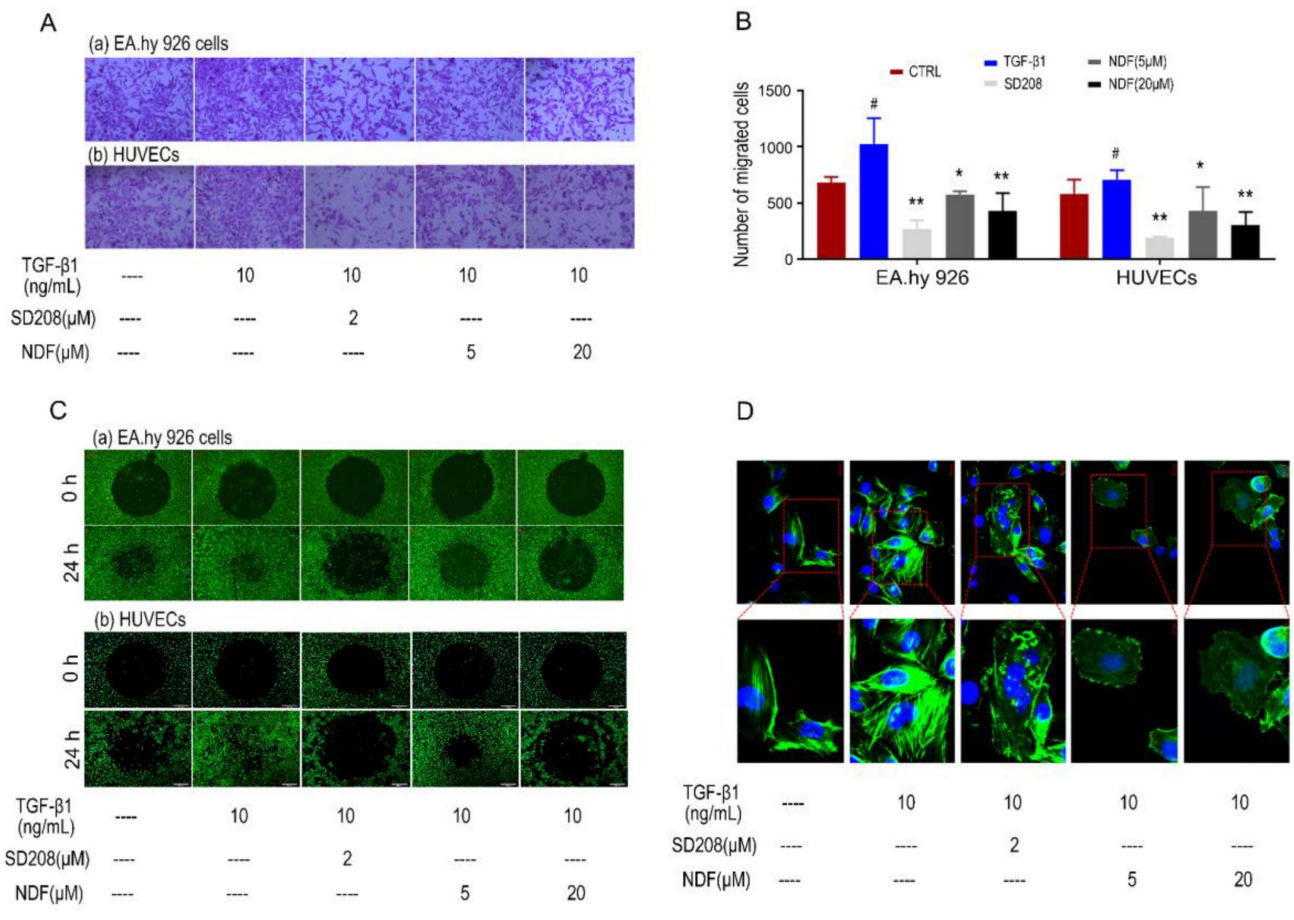
** Effects of NDF on TGF-β1-induced cell motility. A:** Effects of NDF on cell migration (×100); **B:** numbers of migrated cells; **C:** Effects of NDF on cell invasion (×40); **D:** Effects of NDF on F-actin assembly in EA.hy 926 cells (×400 and ×630). *p<0.05 and **p<0.01 compared with TGF-β1; #p<0.05 compared with CTRL.

Using the AP48 chamber system, we initially investigated the effects of NDF on the migration of ECs induced by TGF-β1. Our data revealed a significant decrease in the number of migrated cells in both EA.hy 926 cells and HUEVCs when treated with NDF, compared to the increased migration induced by TGF-β1. (Fig. 2A and 2B). Furthermore, using ORIS^TM^ cell invasion assay system, a dose-dependent inhibition of TGF-β1-induced cell invasion with NDF treatment was observed (Fig. 2C). Additionally, we detected the arrangement of F-actin, a key regulator of cell movement, through phalloidin staining in EA.hy 926 cells. Compared to the control group, TGF-β1 treated cells showed a denser fluorescence intensity and a more regular arrangement of F-actin. In contrast, when the cells were treated with NDF, the expression of F-actin was decreased and F-actin assembly was disrupted and less organized (Fig. 2D).

### Expression of EndoMT biomarkers induced by TGF-β1 can be reversed by NDF

**Figure 3:**
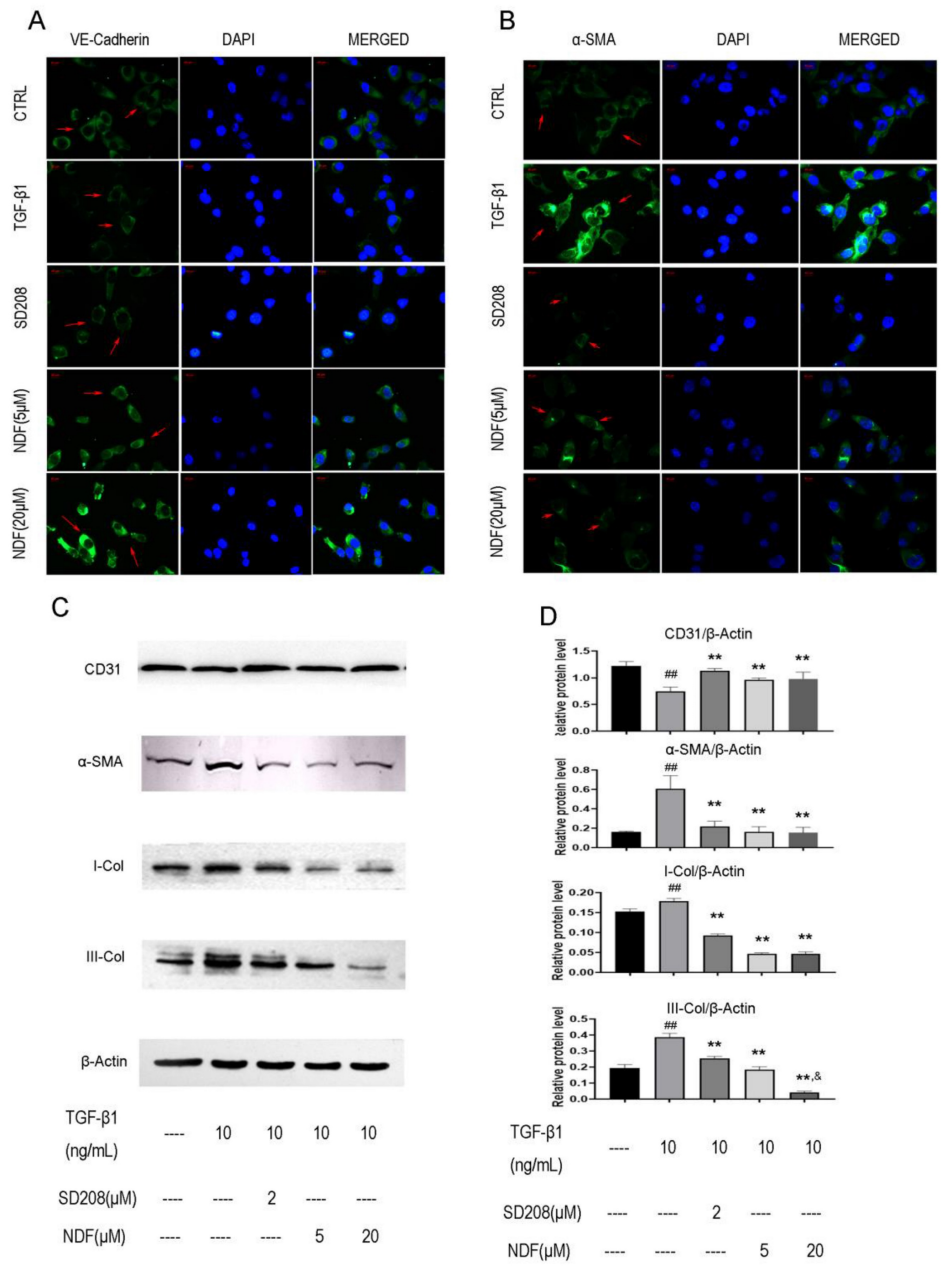
** Effects of NDF on expression of EndoMT markers in EA.hy 926 cells.** Expressions of VE-Cadherin **(panel A)** and α-SMA **(panel B)** were analyzed by immunofluorometric assay (×200). **Panel C and panel D** showed electrophoretic bands and results of densitometric analysis obtained from western blotting assay. **p<0.01 compared with TGF-β1; ##p<0.01 compared with CTRL; &p<0.05 compared with NDF(5μM).

The images obtained from immune fluorescent assay demonstrated a remarkedly decrease of VE-cadherin, a major endothelial adhesion molecule, and a noticeable increase of α-SMA, a typical protein related to mesenchymal functions when EA.hy 926 cells were treated with TGF-β1. However, upon exposure to NDF, the fluorescence intensities of VE-cadherin were increased in a concentration-dependent manner, while the fluorescence intensities of α-SMA were decreased (Fig. 3A and 3B). These results were further confirmed by the utilization of the western blotting assay. Treatment with TGF-β1 resulted in an increase of proteins related to mesenchymal functions, including α-SMA, I-Col and III-Col, and a decrease of CD31, a typical endothelial cells biomarker. Conversely, when the cells were treated with NDF, the expressions of these biomarkers were reversed (Fig. 3C and 3D).

### Destruction of tube-like architecture induced by TGF-β1 can be recovered by NDF

**Figure 4:**
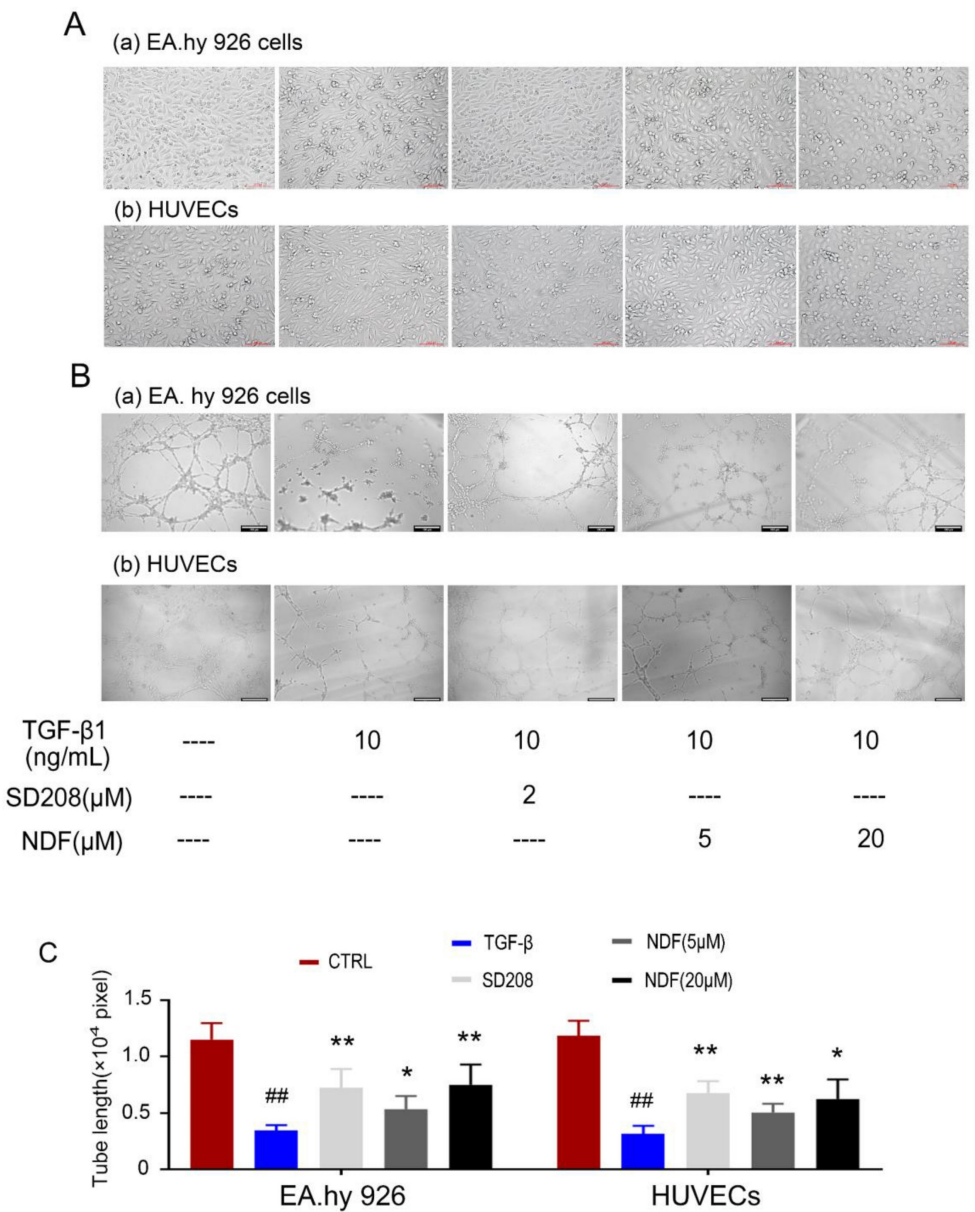
** Effects of NDF on TGF-β1-induced tube-like architecture of ECs. A:** changes of cell morphology; **B:** Effects of NDF on tube formation. **C:** results of total lengths of the completed tubule structure. *p<0.05 and **p<0.01 compared with TGF-β1 group; ##p<0.01 compared with CTRL.

Subsequently, alterations in cellular morphology were observed in both EA.hy 926 cells and HUEVCs. Treatment of endothelial cells (ECs) with 10 ng/mL TGF-β1 resulted in the manifestation of spindle morphology, leading to elongation and slenderization of both ECs. However, these changes were reversed when the cells were incubated with NDF. The original paving stone-like cells appearance were re-revealed in a concentration-dependent manner (Fig. 4A). Additionally, the impact of NDF on tube formation was also evaluated utilizing* in vitro* Matrigel tube formation assay. When the cells were exposed to NDF, the mesh-like structures were re-appeared and the total length of tubular network was dramatically increased compared with TGF-β1 group (Fig. 4B and 4C).

### Phosphorylation of Ezrin can be impaired by NDF

**Figure 5:**
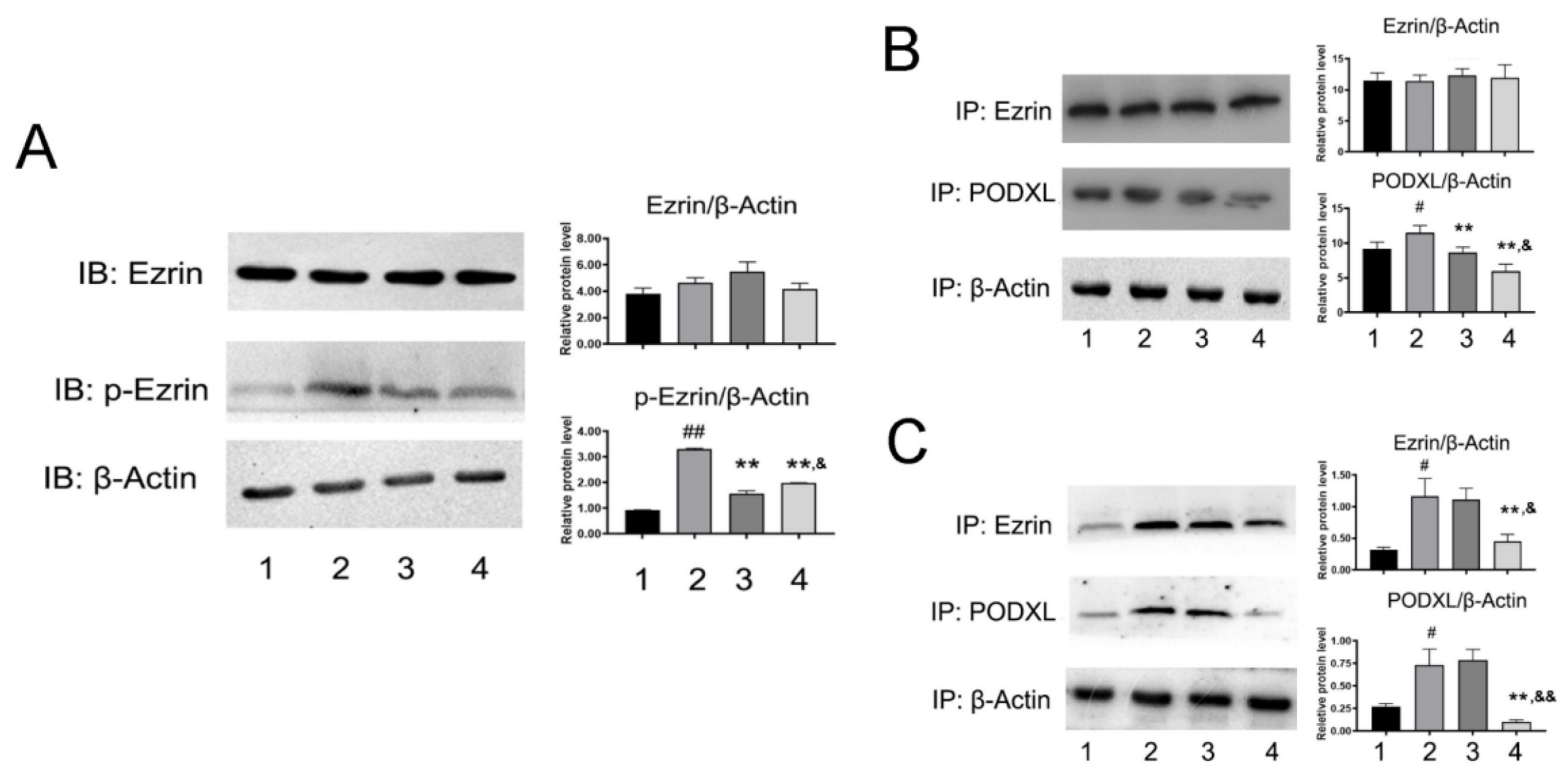
** Effects of NDF on the Ezrin in TGF-β1-treated EA.hy 926 cells. A:** the effects of NDF on expression of Ezrin and p-Ezrin; **B:** Effects of NDF on PODXL-Ezrin interaction by immunoprecipitation while anti-Ezrin antibody was used as sedimental protein; **C:** Effects of NDF on PODXL-Ezrin axis activities by immunoprecipitation while anti-PODXL antibody was used as sedimental protein. *p<0.05 and **p<0.01 compared with control; #p<0.05 and ##p<0.01 compared with TGF-β1; &p<0.05 and &&p<0.01 compared with NDF(5μM). Lane 1: control; lane 2: TGF-β1; lane 3: NDF (5μM); lane 4: NDF (20μM).

Given the important role of Ezrin in cellular motility, we next measured expression and activity of Ezrin. As shown in Fig. 5A, the expression of p-Ezin was significant decreased in NDF-treated cells, but no evident changes were observed in the expression of total Ezrin. Subsequently, the effects of NDF on co-precipitation between Ezrin and PODXL were examined. As shown in Fig. 5B, the content of PODXL in co-sediments was reduced by NDF in a concentration-dependent manner, but no manifest changes in total Ezrin. On the contrary, when the antibody against PODXL was employed in co-immunoprecipitation assay, both contents of Ezrin and PODXL were reduced in NDF-treated cells compared with TGF-β1-treated cells (Fig. 5C). These data suggested that inhibition of Ezrin phosphorylation in TGF-β1-treated ECs may be the crucial step for NDF to impair the process of EndoMT.

### Angiogenesis induced by VEGF can be destroyed by NDF

**Figure 6:**
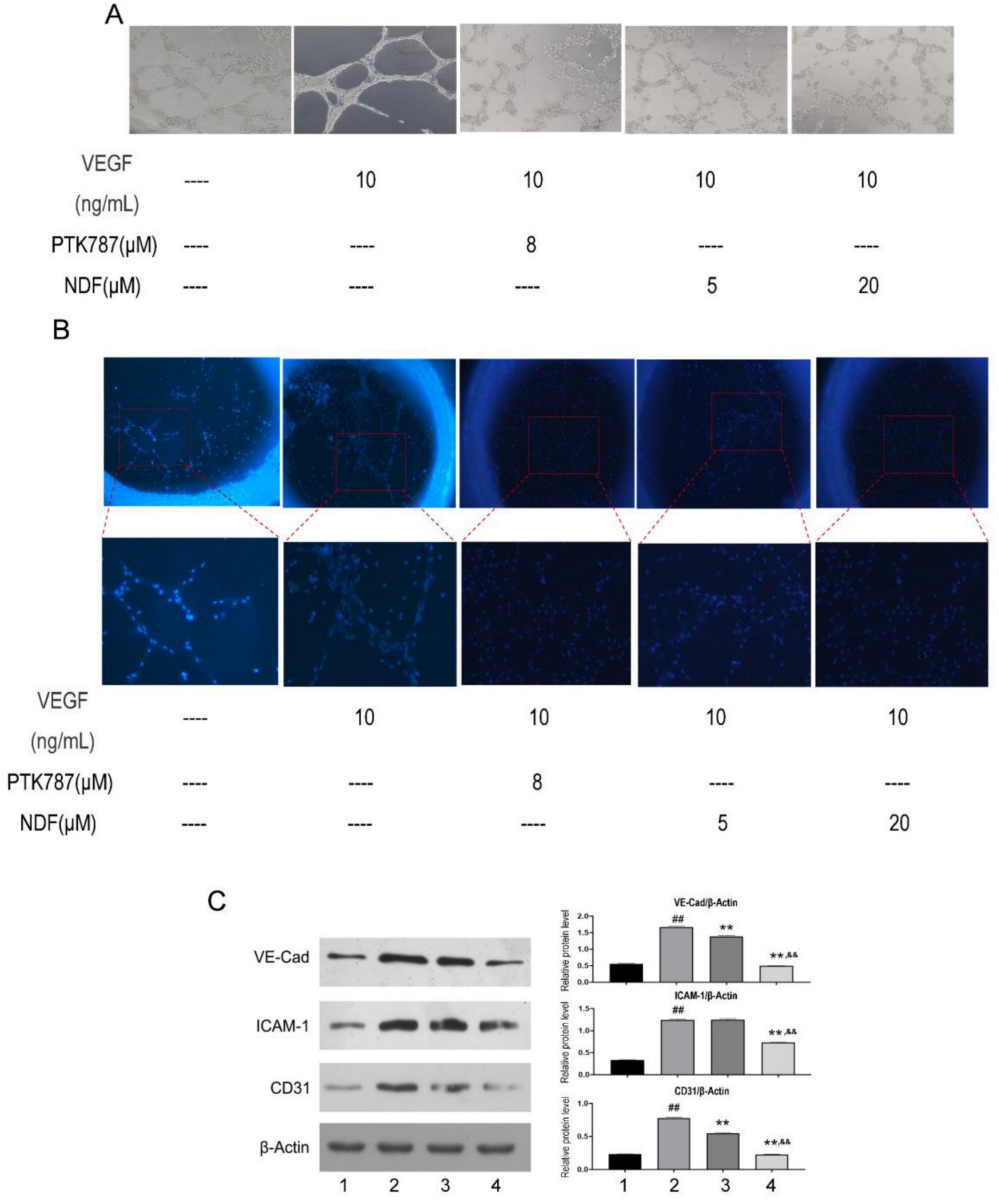
** Effects of NDF on angiogenesis induced by VEGF. A:** Effects of NDF on tube formation of EA.hy 926; **B:** Effects of NDF on rat aortic ring angiogenesis. **C:** Effects of NDF on expression of several angiogenic markers in EA.hy 926 cells. **p<0.01 compared with VEGF group; ##p<0.01 compared with CTRL, &&p<0.01 compared with NDF(5μM).

**Figure 7:**
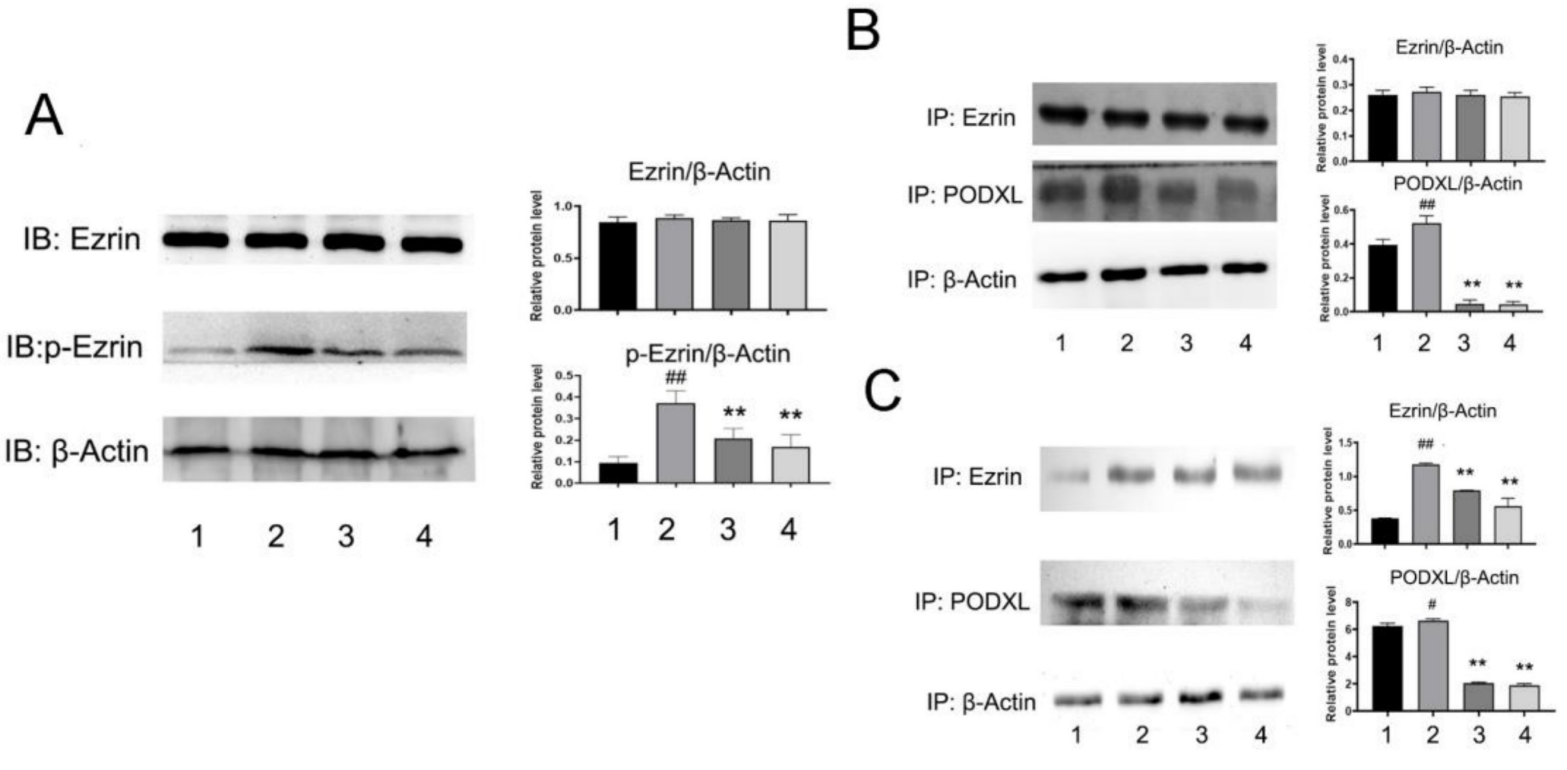
** Effects of NDF on Ezrin in VEGF-treated EA.hy 926 cells. A:** Effects of NDF on expression of Ezrin and p-Ezrin; **B:** Effects of NDF on PODXL-Ezrin interaction by immunoprecipitation while anti-Ezrin antibody was used as sedimental protein; **C:** Effects of NDF on PODXL-Ezrin interaction by immunoprecipitation while anti-PODXL antibody was used as sedimental protein. *p<0.05 and **p<0.01 compared with control; #p<0.05 and ##p<0.01 compared with VEGF. Lane 1: control; lane 2: VEGF; lane 3: NDF (5μM); lane 4: NDF (20μM).

To better understand NDF's effects on angiogenesis, tube formation and rat aortic ring angiogenesis assay were performed. As shown in Fig 6A, The EA.hy 926 cells formed complete mesh-like structures when the cells were incubated with VEGF. But when the cells were exposed to NDF, the tube assembly was destroyed evidently. Additionally, exposure to NDFresulted in significantly attenuation on total numbers of branching and microvessels by rat aortic ring angiogenesis assay (Fig. 6B). Furthermore, the expression levels of several characteristic proteins in ECs, such as VE-cadherin, ICAM-1 and CD31, were significantly decreased (Fig. 6C). It was also found that NDF incubation resulted in suppression of Ezrin phosphorylation and impairment of interaction between Ezrin and PODXL in VEGF-stimulated ECs (Fig. 7A,7B and 7C).

### Inhibition of Ezrin siRNA on cell motility and tube formation can be enhanced by NDF

**Figure 8:**
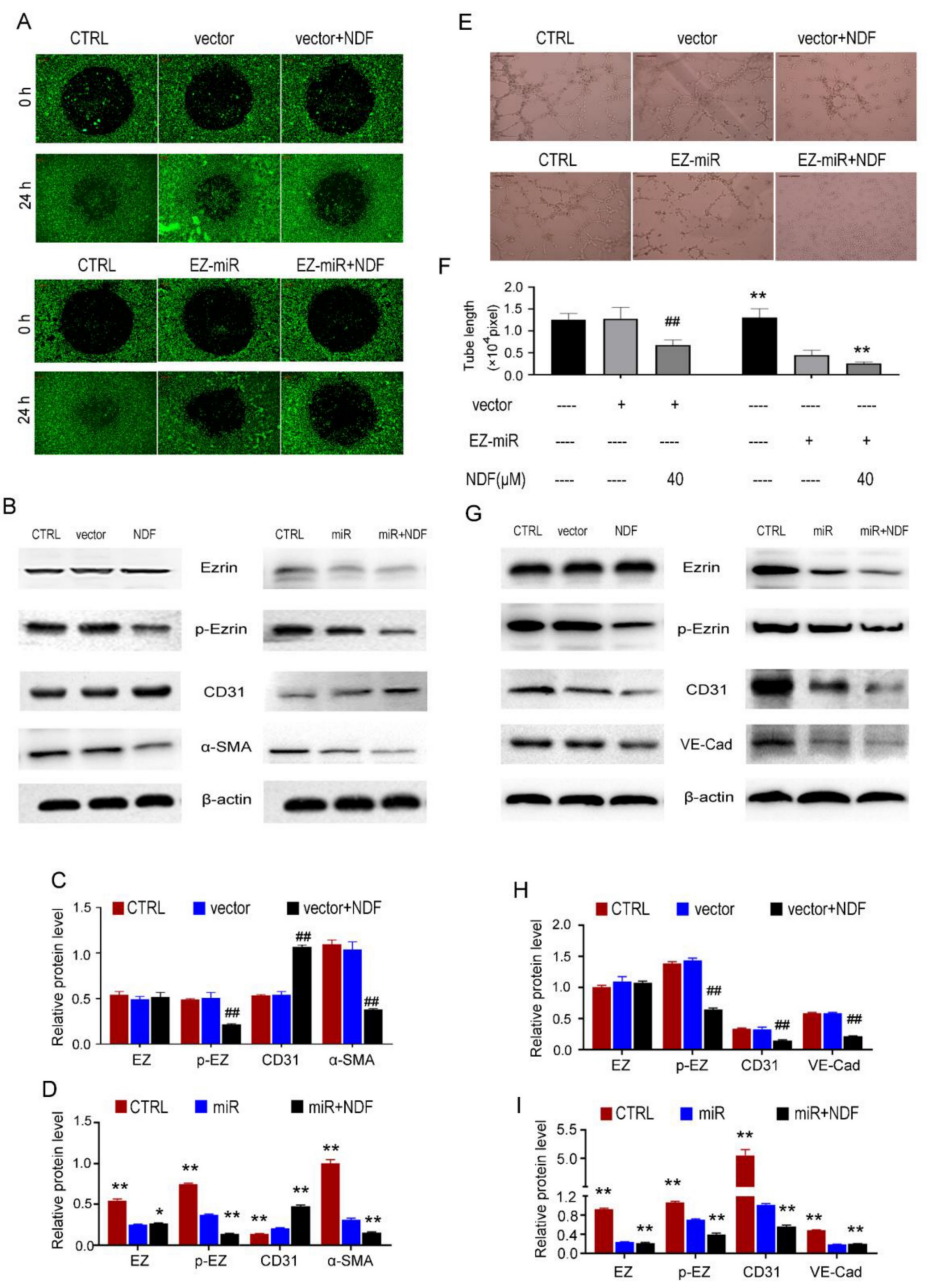
** Effects of NDF on TGF-β1-induced EndoMT and VEGF-induced angiogenesis when Ezrin was knocked-down in EA.hy 926 cells.** Effects of NDF on invasion **(A)** and expression of EndoMT markers **(B, C and D)** in Ezrin-knocking-down EA.hy 926 cells at presence of TGF-β1; Effects of NDF on tube formation **(E and F)**, expression of angiogenic markers **(G, H and I)** in Ezrin-knocking-down EA.hy 926 cells at presence of VEGF. ##p<0.01 compared with CTRL; *p<0.05 and **p<0.01 compared with siRNA.

In order to further substantiate the involvement of Ezrin in the inhibition of EndoMT and angiogenesis mediated by NDF, the technique of RNAi was employed to knockdown Ezrin expression. The findings demonstrated a significant suppression in cell invasion upon silencing Ezrin, and NDF can amplify these inhibitory effects (Fig. 8A). Furthermore, results obtained from Western Blotting analysis also revealed that knockdown of Ezrin led to an increased expression of CD31 and a decreased expression of α-SMA. Furthermore, the inhibitory effects of Ezrin deletion were enhanced by NDF (Fig.8B, 8C and 8D). Similarly, exposure to NDF led to more obvious suppressions on tube formation of EA.hy 926 cells (Fig. 8E and 8F) and on the expression of CD31 and VE-Cadherin compared with transfected cells (Fig. 8G, 8H and 8I).

## Discussion

*Callicarpa nudiflora* Hook is a traditional folk medicine in China used for eliminating stasis to subdue swelling 16. Results from phytochemical studies revealed that the chemical constituents of this herbal medicine include flavonoids, iridoids and phenylpropanoid glycosides etc 17. Several compounds from *Callicarpa nudiflora* have been proved to show anti-inflammatory, haemostasis, hepatitis, and anti-proliferative effects 17. Previously, we focused our interests on searching anti-metastatic leading compounds from this herb, and found that NDF, a secoiridoid glucosides isolated from *Callicarpa nudiflora* 18 showed significant inhibition on TGF-β1-induced migration of EA.hy 926 cells. Since TGF-β1 is an important inducer of EndoMT, we wanted to know whether the inhibitory effect of NDF on TGF-β1-induced migration is associated with its anti-EndoMT effects. In present investigation, we firstly verified the effects of NDF on ECs motility. It was found that NDF suppressed migration, invasion and assembly of F-Actin stimulated by TGF-β1 in both EA.hy 926 cells and HUVECs. These data indicated that NDF may suppress the increased cell motility stimulated by TGF-β1, which may be associated with its inhibition on the F-action arrangement. In addition, results obtained from Western Blotting assay demonstrated that NDF significantly downregulated mesenchymal biomarkers expression and upregulated endothelial markers expression. Furthermore, it was found that NDF can recover the cell shape of ECs and tube-like architecture destroyed by TGF-β1. Taken together, these data demonstrated the inhibitory effect of NDF on EndoMT in terms of cell morphology, cell function and expression of biomarkers.

Ezrin is a principal member of Ezrin/Radixin/Moesin (ERM) family proteins 19. By the phosphorylation of threonine (Thr567) and tyrosine (Tyr353), this protein is activated and binds to membrane proteins through N-terminal domain and F-Actin by C-terminal domain, respectively 20, 21. Therefore, Ezrin is an important structure organizer linking membrane proteins and cytoskeleton and plays important roles in modulating cell motility, adhesion and cell morphology, polarization and EMT 22-24. Led by these concepts, the effects of NDF on Ezrin expression and phosphorylation were evaluated in order to find out whether the inhibitory effects of NDF on EndoMT were related to suppressing expression or/and activation of Ezrin. It was found that NDF significantly downregulated the expression level of p-Ezrin, but no evident changes in total Ezrin expression, which indicated that activation of Ezrin in ECs may be inhibited by NDF. Data from Co-immunoprecipitation assay verified that interaction between Ezrin and PODXL, a binding partner of Ezrin 25 was significantly supressed by NDF. Based on present data, it can be concluded that the inhibitory effects of NDF on TGF-β1-induced EndoMT in ECs may also be associate with its effects on Ezrin phosphorylation.

Aside from the important roles of Ezrin on driving tissue morphogenesis, promoting tumor invasion, EMT and metastasis, the effects of Ezrin on angiogenesis were also paid much attention. Li et. al 23 reported that overexpression of Ezrin led to increase of the vascular mimicry and microtubule formation ability of HUVECs *in vitro*, and upregulatory expression of VEGF and HIF1α* in vivo*. In contrast, in the Ezrin depletion cells, the vascular mimicry and microtubule formation ability of HUVECs were reduced. The results from Zhao's study also suggested that Ezrin may have pro-angiogenic properties in cancer development 26. Therefore, we then assessed the effects of NDF on VEGF-induced angiogenesis. The data showed that NDF destroyed mesh-like structure induced by VEGF and obviously impaired Ezrin phosphorylation and Ezrin-PODXL interaction. These data indicated that NDF inhibited TGF-β1 induced EndoMT and VEGF stimulated angiogenesis on ECs, which may be associated with its inhibitory effects on Ezrin phosphorylation.

However, some researchers have proposed that, besides inducing complete mesenchymal transition of ECs, EndoMT process may drive ECs only partially and reversible transition, and there is a growing body of evidence to suggest that sprouting angiogenesis may be a typical partial EndoMT^27^, ^28^. During partial EndoMT, ECs acquired mesenchymal phenotypes, including increased cell motility, destabilization of cell-cell junctions and appearance of numerous filopodia, while some of their original features were still preserved, such as maintenance of junctional contacts which ensured these angiogenic cells did not detach from their neighbours. On the other hand, it was also found that several transcriptional factors found in EndoMT process, such as Snail, Slug and Zeb1, were also significantly induced during sprouting angiogenic process, which may further demonstrate that angiogenesis is a partial EndoMT event 29, 30. However, in our experimental design, two different inducers, TGF-β1 and VEGF, were respectively used to induce EndoMT and angiogenesis. Hence, whether ECs underwent a partial EndoMT was still uncertain at our assessed timepoint. Therefore, it was tough to conclude that NDF showed inhibitory effects on partial EndoMT. Taken together, we concluded, based on current data, NDF exerts dual effects on both EndoMT and angiogenesis.

In aggregate, our data suggested that NDF exerted dual suppression on TGF-β1-induced EndoMT and VEGF-induced angiogenesis, which may be associated with reduction of Ezrin activities. But the more proofs are still needed to further elucidate the exact impacts of NDF on EndoMT and angiogenesis, such as *in vivo* experiment. In addition, the effects of NDF on partial EndoMT and the mechanism of NDF on Ezrin phosphorylation will also be involved in the future studies.

## Abbreviations

α-SMA: α-smooth muscle actin
BCA: Bicinchoninic Acid Assay
BMP: bone morphogenetic protein
BSA: Bovine serum albumin
CAFs: cancer associated fibroblasts
CBS: cytoskeleton buffer with sucrose
DAPI: 4′,6-diamidino-2-phenylindole
DMEM: Dulbecco's Modified Eagle's Medium
ECs: endothelial cells
EMT: epithelial-mesenchymal transition
EndoMT: Endothelial to mesenchymal transition
F-actin: filamentous actin
FBS: fetal bovine serum
FSP-1: fibroblast-specific protein-1
HUVECs: Human Umbilical Vein Endothelial Cells
ICAM-1: intercellular adhesion molecule-1
I-Col: I-Collagen
III-Col: III-Collagen
N-Cadherin: Neural-cadherin
NDF: nudifloside
PAI-1: Plasminogen Activator Inhibitor 1
PBS: phosphate buffer saline
PECAM-1: platelet endothelial cell adhesion molecule-1
PODXL: Podocalyxin
PVDF: Polyvinylidene fluoride
RIPA: Radioimmunoprecipitation assay buffer
siRNA: small interfering RNA
siRNA: Small interfering RNA
SM22a: Smooth Muscle Protein 22-α
TGF-β1: transforming growth factor-β1
TGF-βRI: TGF-β Receptor inhibitor
TIE1: tyrosine kinase with immunoglobulin-like and EGF-like domains 1
TIE2: tyrosine kinase with immunoglobulin-like and EGF-like domains 2
TME: tumor microenvironment
VE-cadherin: vascular endothelial cadherin
VEGF: vascular endothelial growth factor
vWF: von Willebrand Factor
